# Treatment of mid-shaft clavicle fractures: A comparative study

**DOI:** 10.4103/0973-6042.57895

**Published:** 2009

**Authors:** David S. Thyagarajan, Marion Day, Colin Dent, Rhys Williams, Richard Evans

**Affiliations:** Department of Trauma and Orthopaedics, University Hospital of Wales, Cardiff, South Wales

**Keywords:** Clavicle, midshaft clavicle fractures, clavicle pins

## Abstract

We retrospectively evaluated 51 patients (17 in each of three groups) with mid shaft clavicle fractures. Group 1 underwent intramedullary stabilization using clavicle pins. Group 2 underwent open reduction and internal fixation using plates and group 3 underwent non operative treatment with a sling. Group1 patients progressed to union within 8 to 12 weeks. In Group 2, six patients had scar related pain and two had prominent metal work and discomfort and in group 3, three patients developed non union and one had symptomatic malunion. Our results suggest that the displaced and shortened midshaft clavicle fractures require operative fixation and the techniques of clavicle pinning resulted in less complications, short hospital stay and good functional outcome.

## INTRODUCTION

Fractures of the clavicle account for 44% of injuries around the shoulder girdle,[1] approximately 70% to 80% of which occur in the mid third.[2] Displaced and shortened fractures of the mid third of the clavicle are common in young, athletic populations and are frequently high-energy injuries sustained in road traffic accidents or sports injuries. It is this subgroup of patients, viz., those with displaced and shortened mid-shaft fractures of the clavicle, that often requires operative fixation.[3] Closed treatment for displaced middle-third fractures gives poor results.[4] Several techniques of fixation have been described in literature, including the use of plates, Kirschner wires,[5] Steinman pins,[6] external fixators[7,8] and even plaster constructs.[9] Previously used intramedullary devices were smooth and hence lacked compression at the fracture site. The aim of this study was to compare the outcome achieved by three groups of patients treated for displaced and shortened mid-clavicle fractures.

## MATERIALS AND METHODS

We retrospectively evaluated 51 consecutive patients (three groups, with 17 patients in each) that had sustained mid-shaft clavicle fractures with 100% displacement and more than 2 cm shortening. They had all been treated in a single large teaching hospital.

**Figure 1:**
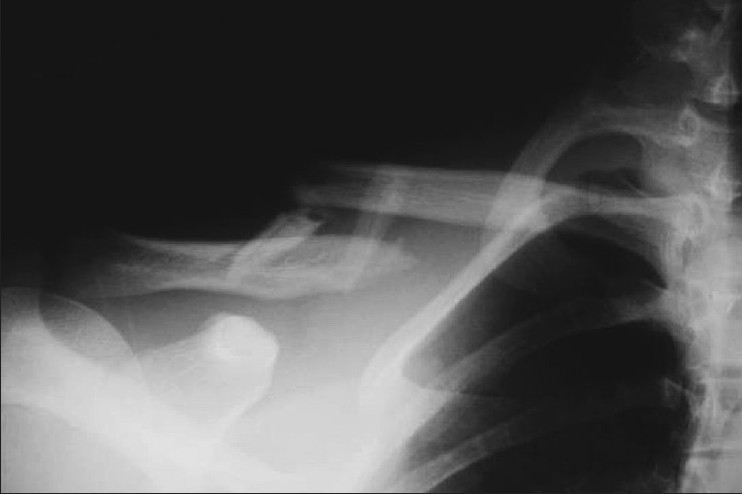
The radiograph showing 100% displacement and more than 2 cm shortening of mid-shaft clavicle fracture

Only patients with mid-shaft clavicle fracture with 100% displacement and more than 2 cm shortening were included in the study [Figure 1]. Eighteen out of the 51 mid-clavicle fractures in our study had communition at the fracture site with a butterfly fragment. Seven were in the clavicle pin group; 6, in the plating group; and 5, in the conservative treatment group. One patient in the nonoperative group had contralateral extra-articular wrist fracture, which was treated in plaster for 6 weeks. There was no case of floating shoulder in any of the three groups in the study.

**Figure 2:**
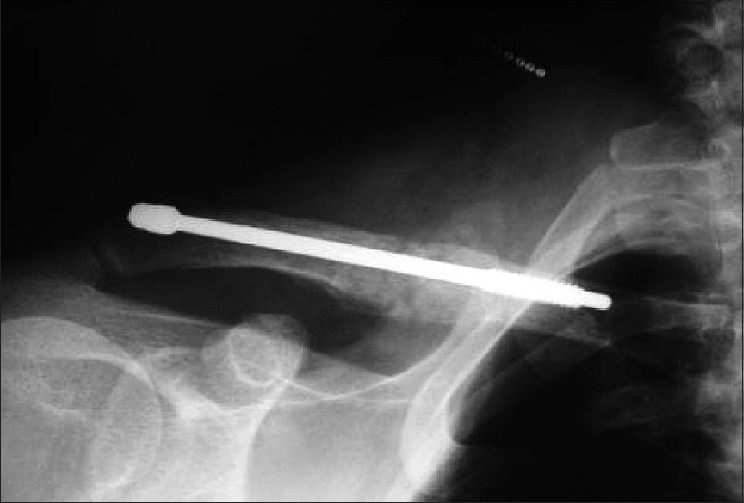
Radiograph showing intramedullary stabilization using rockwood clavicle pins

Patients in group I underwent intramedullary stabilization using clavicle pins (Rockwood Clavicle Pins, Depuy) [Figure 2]. There were 16 males and 1 female, with an average age of 28 years (range, 15-56 years). Eleven (65%) of the fractures in this group were sustained following a sports injury; 2 (12%), following road traffic accident; 2 (12%), after a direct fall onto the shoulder; and 2 (12%), following a fall on an outstretched hand.

Patients in group II underwent plating using LC-DCP (low-contact dynamic compression plate). There were 15 males and 2 females, and the average age was 32.1 years (range, 17-46 years). Eight (47%) fractures were sustained following a sports injury; 4 (24%), after a road traffic accident; 3 (18%), following a fall on an outstretched hand; and 2 (12%) following a direct fall onto the shoulder. One patient in this group had a grade one compound injury.

Patients in group III had nonoperative treatment with a broad arm sling and mobilization. There were 12 males and 5 females, with an average age of 34.5 years (range, 17-64 years).

Four (24%) fractures in this group were sustained following a sports injury; 4 (24%), after a fall on an outstretched hand; 3 (18%), after a road traffic accident; 3 (18%), following an assault; and 3 (18%), following a direct fall onto the shoulder. In groups I and II, the time interval between the occurrence of fracture and the operation ranged from 4 to 20 days (average, 10.3 days).

All patients were assessed clinically and radiologically. The functional outcome was assessed using the American Shoulder and Elbow Surgeons (ASES) score and Constant score. The average follow-up period was 5.9 months (range, 4-11 months). The patients in group I that underwent the procedure using Rockwood clavicle pins were assessed functionally after removal of the pin.

As the clavicle pin was a new device, during the early stages, some of the teams within the department were treating displaced mid-clavicle fractures with plates or were providing nonoperative treatment. Hence we were able to get three groups for this study from the same institution.

### Technique of intramedullary fixation

**Figure 3:**
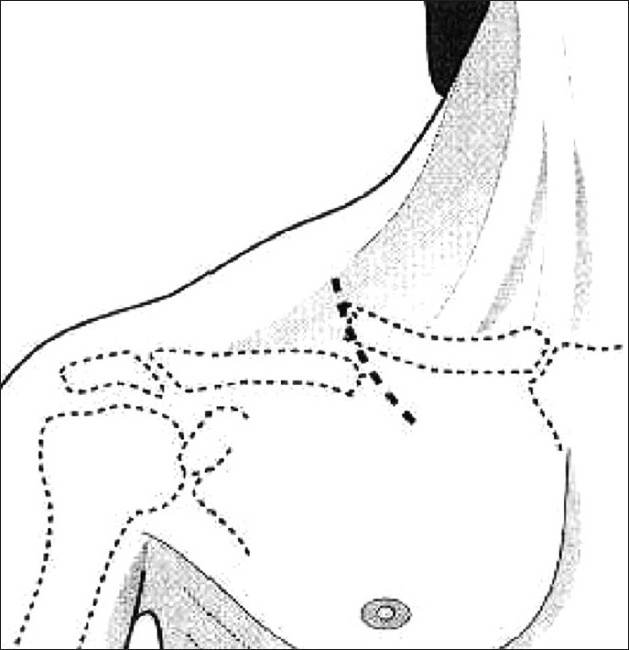
Diagram showing the site for incision

The technique of intramedullary fixation is similar to the original technique described by Boehme *et al*.[10] The patient is placed in a modified beach chair position with a surgical headrest. A 2-3 cm incision is made over the fracture site in the direction of Langer's lines [Figure 3]. With minimal soft tissue dissection, the fracture site is identified. Care should be taken to prevent injury to the middle branch of the supraclavicular nerve, which is usually found beneath the platysma muscle near the mid-clavicle. The small butterfly fragment is usually found anteriorly and is left attached to the soft tissue.

Elevate the proximal end of the medial fragment of the clavicle through the wound using a bone-holding clamp. The medullary canal is sized using the drills available in the set. Then drill and tap the medullary canal manually with a Jacobs's chuck and T handle, taking care not to penetrate the anterior cortex. Image intensifier is used to check the orientation of the drill. Next, elevate the lateral fragment through the wound and drill the medullary canal manually using the same-sized drill under image intensifier guidance until it is out through the posterolateral cortex of the clavicle just posterior to the acromioclavicular joint. Then tap the intramedullary canal of the lateral fragment.

Now, remove the nuts from the clavicle pin assembly and introduce the trocar end of the clavicle pin into the canal of the lateral fragment from the medial end and advance it until it exits through the previously drilled hole in the posterolateral cortex and through the skin, for which, make a small stab incision. Attach the Jacob's chuck and the T handle to the exiting clavicle pin tip and withdraw so that the pin advances into the lateral fragment. Now, reduce the fracture and advance the pin into the medial fragment. Insert the medial nut followed by the lateral nut in the clavicle pin and cold-weld the two nuts by tightening the lateral nut against the medial nut. Then, using the T handle, advance the clavicle pin into the medial fragment until it comes in contact with the anterior cortex of the medial fragment. Check this position with the image intensifier. Now, break the cold weld between the nuts by turning the lateral nut counterclockwise and then advance the medial nut until it is against the lateral cortex of the clavicle. Then tighten the lateral nut until it engages the medial nut. Cut the pin at the lateral nut using a pin cutter (Surgical Technique, Depuy manual).

Postoperatively no immobilization is required. The patient can return to full daily activities as soon as tolerated. Forward flexion is limited to 90 degrees for 3-4 weeks due to the fact that movements beyond 90 degrees can cause rotation of the fracture fragments and the pin, leading to irritation of the soft tissue at the posterolateral wound. Pin removal is recommended after 12 weeks post-op following radiological union.

### Plating

The plating in group II patients was done through the standard approach using 3.5-mm LC-DCP plates with at least three bicortical screws on either side of the fracture.

## RESULTS

Group I: In patients that underwent clavicle pin stabilization, radiological union was apparent by 8 to 12 weeks. There were two superficial wound infections at the posterolateral wound site, both of which healed with oral antibiotics. One of these patients had prominent metal work at that site. There were no scar-related symptoms, and all patients were satisfied cosmetically. The average Constant score was 97.8, and the average ASES score was 99.6 [Table 1].

**Table 1:** Distribution of ASES and constant scores by groups

Variables	Group I clavicle pin (*n* = 17)			Group II plating (*n* = 17)			Group III nonoperative (*n* = 17)			Kruskal-Wallis test *P* value
	Mean	SD	Median	Mean	SD	Median	Mean	SD	Median	
ASES score – subjective										
Pain	9.9	0.2	10.0	9.1	1.1	9.0	9.1	2.0	10.0	0.015
Activities	29.5	0.9	30.0	28.0	1.9	29.0	27.2	6.0	29.0	0.010
ASES score – objective										
Range of motion	40.0	0	40.0	39.9	0.5	40.0	38.2	4.6	40.0	0.006
Strength	20.0	0	20.0	20.0	0	20.0	19.9	0.5	20.0	0.368
Total score overall – score	99.6	0.7	100.0	97.0	2.9	98.0	94.4	12.6	98.0	0.001
Constant score – subjective	34.3	1.0	35.0	32.2	1.9	33.0	32.2	4.8	34.0	0.001
Constant score – objective	63.5	2.3	65.0	61.4	3.5	60.0	56.8	11.9	60.0	0.021
Total score overall – constant	97.8	2.5	99.0	93.7	4.4	94.0	89.0	16.0	94.0	0.002

Group II: In patients that underwent plating of the clavicle, there was one superficial wound infection which healed well with oral antibiotics and one deep wound infection requiring surgical wound debridement in theater on two occasions under general anesthesia. There were no predisposing factors for infection found in these cases. The grade one open fracture in this group progressed towards union with no wound problems and a good functional outcome. One patient had nonunion, 2 had delayed union. Six (35%) patients in this group had scar-related pain, and 4 (24%) had numbness distal to the scar. Two (12%) patients had prominent metal work causing discomfort, and they underwent removal of hardware a year following the fixation. One patient was cosmetically unhappy. The average Constant score was 93.7, and the average ASES score was 97.0.

Group III: In patients treated conservatively, 3 (18%) developed a nonunion which required operative intervention. One patient developed a symptomatic malunion, 1 had symptoms of brachial plexus irritation and 4 (24%) were cosmetically unhappy. The average Constant score was 89.0, and the average ASES score was 94.0.

### Statistical analysis

The statistical analysis was performed using the Kruskal-Wallis one-way analysis of variance by ranks. The results were significant at 5% level [Table 2].

**Table 2:** Comparison of ASES and constant scores between the three groups

Variables	Mean rank			
	Group I	Group II	Group III
ASES score - subjective			
Pain	31.7	20.1	26.2
Activities	34.0	19.6	24.4
ASES score – objective			
Range of motion	29.5	28.1	20.4
Strength	26.5	26.5	25.0
Total score overall – score	26.5	19.9	21.6
Constant score – subjective	34.5	16.8	26.4
Constant score – objective	33.1	24.6	20.3
Total score overall – constant	36.5	20.6	21.0

In summary, 4 (23.5%) out of the 17 patients treated conservatively required surgical intervention (3 nonunion and 1 symptomatic malunion). Six (35.2%) out of the 17 patients in the plating group had scar-related symptoms. Seven (41%) out of the 17 patients in the conservative group and 6 (35%) out of the 17 patients in the plating group had pain during daily activities. Only 2 (12%) out of the 17 patients in the clavicle pin group had mild pain. Four (23.5%) of the 17 patients in the conservative group and 4 (23.5%) of the 17 patients in the plating group had pain on lying on the affected side. Two (12%) out of the 17 patients in the conservative group could not return to their heavy manual work due to the symptoms from nonunion. The clavicle pin fixation was performed as a day surgery as was their removal. The plating group required 2 to 3 days' hospital stay.

## DISCUSSION

The clavicle fracture represents 5% to 15% of all fractures. As many as 80% of clavicle fractures occur in the middle third. Many of these fractures occur in young and athletic individuals. Neer reported a nonunion rate of 0.1%,[11] and Rowe reported a nonunion rate of 0.8%[2] in conservatively treated clavicle fractures. No one so far has been able to reproduce these results. In literature, more recent studies have shown an incidence of nonunion in conservatively treated clavicle fractures of between 10% and 15%. Hill *et al*. reported a 15% nonunion rate and 31% patient dissatisfaction rate in conservatively treated displaced middle-third fractures of the clavicle.[4] Fractures of the mid-shaft clavicle with a shortening of more than 2 cm predispose to nonunion.[12] Rockwood *et al*. showed that when a nonunion occurs, it almost always involves the middle third of the clavicle. In his series, 80% of nonunion occurred in the displaced and shortened mid-shaft clavicle fractures.[10] Prospective observational cohort study by Robinson *et al*. found a significantly higher nonunion rate (21%) for displaced communited mid-shaft clavicle fractures treated nonoperatively.[13] Nowak *et al*. reported that 46% of the patients with clavicle fracture in his study had persistent symptoms 10 years after the injury despite the fact that only 7% developed nonunion.[14] In our study, 41% (7/17) of the patients in the nonoperative group had pain during activities. Two out of the 7 patients had severe pain due to the nonunion. There were 3 (17.6%) cases of nonunion in the nonoperative group.

Fractures of the mid-shaft clavicle with 100% displacement and more than 2 cm shortening require surgical fixation. Various operative techniques have been tried in the past. Plates-and-screws fixation is the most commonly used option by many shoulder surgeons. However, plates and screws require significant soft tissue stripping, which may compromise the blood supply to the clavicle and interfere with subsequent healing. The bicortical screws on the clavicle may act as multiple stress raisers leading to fractures. Bostman *et al*. studied 103 patients treated with open reduction and internal fixation using plates; among those patients, 43% had complications; 15%, major complications; and 14% required re-operation.[15] However, more recent randomized clinical studies comparing plate fixation with nonoperative treatment for mid-clavicle fractures have shown that 2 (3.2%) out of the 62 patients in the plating group and 7 (14.2%) out of the 49 patients in the nonoperative group developed nonunion. The wound infection rate in their study was 4.8% (3/62), and they were managed with antibiotics and local wound care and subsequently underwent removal of metal work once the fracture had healed.[16]

In our study, 1 patient in the plating group developed deep wound infection that required surgical debridement on two occasions in theater. The patient subsequently developed delayed union; scar-related symptoms, including numbness distal to the scar; moderate pain during activities; with a Constant and ASES scores of 81 and 91, respectively. As many as 35% (6/17) of the patients in the plating group had pain during activities, and 3 out of them had moderate pain.

External fixators have been used in open fractures and nonunions of mid-shaft clavicle.[8] Pin track infection is common and many patients resent the devices.[17]

Previously used intramedullary devices were smooth and hence lacked compression at the fracture site and therefore migrated.[18] The new-generation intramedullary device (Rockwood Clavicle Pins, Depuy) is designed with a differential pitch and hence allows compression at the fracture site and minimizes hardware migration. It requires a very small incision at the fracture site with minimal soft tissue dissection and hence avoids the risk of impaired blood supply to the bone.

With conservative treatment for displaced and shortened mid-shaft clavicle fractures, we are not meeting the expectations of our patients. In our study, 23.5% (4/17) of the patients initially treated conservatively required operative treatment. As many as 41% of the patients in the conservative group had pain during daily activities.

Our study has limitations. This was a small retrospective study with potential for a type II error. If there are instability in the system, would not the risk of nonunion have been higher? A randomized controlled trial with a larger sample size is required in future to confirm the outcome achieved in our study by the three groups of patients treated for displaced and shortened mid-clavicle fractures.

The technique of clavicle pinning resulted in minimal complications, short hospital stay and excellent functional outcomes. From our experience, we would recommend the use of clavicle pins for displaced and shortened mid-shaft clavicle fractures.
